# Intercellular Trafficking of Gold Nanostars in Uveal Melanoma Cells for Plasmonic Photothermal Therapy

**DOI:** 10.3390/nano10030590

**Published:** 2020-03-24

**Authors:** Rubén Ahijado-Guzmán, Natalia Sánchez-Arribas, María Martínez-Negro, Guillermo González-Rubio, María Santiago-Varela, María Pardo, Antonio Piñeiro, Iván López-Montero, Elena Junquera, Andrés Guerrero-Martínez

**Affiliations:** 1 Departamento de Química Física, Universidad Complutense de Madrid, Avenida Complutense s/n, 28040 Madrid, Spain; natsanch@ucm.es (N.S.-A.); mmnegro@ucm.es (M.M.-N.); ggrubio@ucm.es (G.G.-R.); ivanlopez@quim.ucm.es (I.L.-M.); junquera@quim.ucm.es (E.J.); 2 Instituto de Investigación Sanitaria de Santiago (IDIS), Xerencia de Xestión Integrada de Santiago (XXIS/SERGAS), Hospital Clínico Universitario de Santiago, Travesía da Choupana s/n, 15706 Santiago de Compostela, Spain; mariasantiagovarela@hotmail.com (M.S.-V.); maruxapardo@hotmail.com (M.P.); anpices@gmail.com (A.P.); 3 Instituto de Investigación Sanitaria Hospital 12 de Octubre (imas12), Avda. Córdoba s/n, 28041 Madrid, Spain

**Keywords:** gold nanostars, nanoparticle endocytosis, nanoparticle exocytosis, femtosecond pulse laser, plasmonic photothermal therapy

## Abstract

Efficient plasmonic photothermal therapies (PPTTs) using non-harmful pulse laser irradiation at the near-infrared (NIR) are a highly sought goal in nanomedicine. These therapies rely on the use of plasmonic nanostructures to kill cancer cells while minimizing the applied laser power density. Cancer cells have an unsettled capacity to uptake, retain, release, and re-uptake gold nanoparticles, thus offering enormous versatility for research. In this work, we have studied such cell capabilities for nanoparticle trafficking and its impact on the effect of photothermal treatments. As our model system, we chose uveal (eye) melanoma cells, since laser-assisted eye surgery is routinely used to treat glaucoma and cataracts, or vision correction in refractive surgery. As nanostructure, we selected gold nanostars (Au NSs) due to their high photothermal efficiency at the near-infrared (NIR) region of the electromagnetic spectrum. We first investigated the photothermal effect on the basis of the dilution of Au NSs induced by cell division. Using this approach, we obtained high PPTT efficiency after several cell division cycles at an initial low Au NS concentration (pM regime). Subsequently, we evaluated the photothermal effect on account of cell division upon mixing Au NS-loaded and non-loaded cells. Upon such mixing, we observed trafficking of Au NSs between loaded and non-loaded cells, thus achieving effective PPTT after several division cycles under low irradiation conditions (below the maximum permissible exposure threshold of skin). Our study reveals the ability of uveal melanoma cells to release and re-uptake Au NSs that maintain their plasmonic photothermal properties throughout several cell division cycles and re-uptake. This approach may be readily extrapolated to real tissue and even to treat in situ the eye tumor itself. We believe that our method can potentially be used as co-therapy to disperse plasmonic gold nanostructures across affected tissues, thus increasing the effectiveness of classic PPTT.

## 1. Introduction

The production and application of plasmonic gold nanoparticles (Au NPs) for medical purposes have notably expanded in the last decade due to their remarkable optical properties and high biocompatibility [1,2,3]. In nanomedicine, gold nanostars (Au NSs) have emerged as promising Au NPs owing to their strong absorption-to-scattering ratio compared to that of, for example, spherical Au NPs with similar sizes [4,5]. Moreover, Au NSs are attracting further attention on account of their tunable localized surface plasmon resonance (LSPR) response, which can be readily shifted to the near-infrared (NIR) region of the electromagnetic spectrum [6,7]. These optical features, located within the optical window of biological tissue (650–1000 nm) [8,9], make Au NSs particularly suitable for in vitro and in vivo applications, such as potential materials for plasmonic photothermal therapy (PPTT) [10,11,12].

PPTT employs cell-internalized Au NPs to turn non-harmful laser light into thermal energy resulting from the interaction of laser radiation and the LSPR of the plasmonic nanostructures [13]. The so-generated intracellular heating has shown high PPTT efficiency for killing cancer cells through apoptosis [14] and/or photothermolysis [11] processes. In respect to the morphology of the nanocrystals, the strong subwavelength confinement of light near the boundary of anisotropic Au NPs has been effectively used to induce cell death [3,4,15], suggesting the potential application of such systems in cancer treatment. In this sense, Au NSs with highly morphological anisotropy have been successfully employed for the enhancement of PPTT [12].

Regarding the laser source, under continuous wave (CW) laser irradiation, the overall effect of mild thermal heating on Au NSs is intense enough to induce the apoptosis of cancer cells [11]. Complementary to CW sources [16], pulse lasers enable irradiation with low fluences in ultrashort periods of time, allowing PPTT effects through the highly localized heating of intracellular Au NPs [15]. Interestingly, the maximum permissible exposure (MPE) threshold of skin using pulse lasers (0.4 W/cm^2^ at 850 nm) [17] is often exceeded using spherical Au NPs [16]. However, there are examples of effective PPTT in cancer cells using Au NSs [12] or Au nanorod assemblies [18] under excitation with a NIR femtosecond (fs) laser at a power density of ~0.2 W/cm^2^, which is below the required MPE threshold of skin.

The cellular response to Au NPs can be potentially exploited as a tool for alternative cancer therapies. In general, cells, due to their unsettled capacity to uptake, retain, release, and re-uptake Au NPs, offer enormous opportunities for research by varying the cell line characteristics and the Au NP size, shape, and surface modification [19,20,21]. While a few studies exist on the ability for re-endocytosis of Au NPs and their dilution due to cell division [22,23], the consequences of intercellular trafficking during PPTT remain unclear [22]. Additionally, uveal malignant melanoma is the most common primary intraocular tumor in adults. Currently, the most commonly used local treatments for this tumor are brachytherapy (iodine-125, ruthenium-106, or palladium-103, or cobalt-60) and teletherapy (proton beam, helium ion, or stereotactic radiosurgery using a cyber knife, gamma knife, or linear accelerator); the alternative to radiation is eye enucleation [24,25]. In this context, uveal melanoma cells offer an excellent biological environment to study due to the ubiquitous use of NIR CW and pulse lasers in multiple types of eye surgery [26,27,28].

Inspired by these precedents, we have tackled the in vitro study of PPTT in uveal melanoma cells loaded with Au NSs under irradiation with a fs pulse laser (800 nm Ti:sapphire 90 fs laser pulses, 80 MHz) by two different approaches. On the one hand, we have investigated the photothermal effect associated with the dilution of Au NSs generated during cell division. The use of Au NSs with the LSPR band centered at 800 nm, combined with low pulse laser irradiation at the NIR (0.21 W/cm^2^, below the MPE threshold of skin), afforded high PPTT efficiency after three and four cell-division cycles at Au NS concentrations of 2 pM and 8 pM, respectively. On the other hand, we have examined the PPTT response upon cell division and in the presence of exocytosis/re-endocytosis processes by mixing Au NS-loaded and non-loaded cells. At an effective Au NS concentration of 4 pM, we observed trafficking of Au NSs between loaded and non-loaded cells, resulting in effective PPTT under optimal low irradiance conditions, even after four division cycles. Therefore, our study reveals the ability of these cells to release and re-uptake Au NSs that retain their unique photothermal properties throughout several cell division cycles. These findings can potentially be used as an alternative co-therapy to disperse plasmonically active gold nanostructures across affected tissues in situ or the eye itself, thus increasing the effectiveness of classic PPTT.

## 2. Results and Discussion

**Figure 1:**
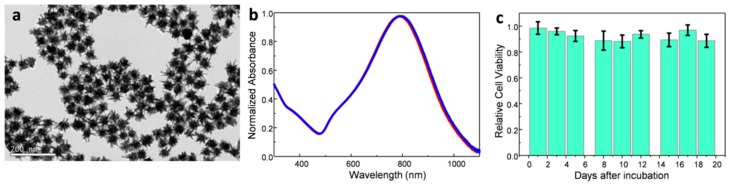
(**a**) TEM image of the synthetized Au NSs. (**b**) UV-vis-near-infrared (NIR) spectra of the gold nanostars (Au NS) colloidal solution with the localized surface plasmon resonance (LSPR) band at ca. 800 nm (red) and at 795 nm (blue), before and after transfer to physiological conditions, respectively. (**c**) Cell viability test after 12 h of incubation of Au NSs with uvea cells (3 independent samples were tested and averaged; the error bars show the standard deviation of the relative cell viability). The cell viability shows cell survival above 90% after 19 days.

Plasmonic Au NSs with sizes of 50.2 ± 4.0 nm analyzed by transmission electron microscopy TEM (Figure 1a), and LSPR band centered at ca. 800 nm (Figure 1b), were synthesized by a colloidal seed-mediated growth method (see Materials and Methods Section) [29]. The synthesis was based on the preparation of citrate seeds of 14.0 ± 1.0 nm, and a subsequent growing and branching step in which Au NSs (56 ± 6 nm measured by dynamic light scattering; zeta potential of −21 ± 1 mV in good agreement with previous results [30]) functionalized with a thiol-modified polyethylene glycol (PEG-SH, 6 kDa) were obtained [31], in order to prevent aggregation at physiological ionic strengths (Figure 1b) and provide excellent biocompatibility [32]. To test the cytotoxicity of the Au NSs, uveal melanoma cells were incubated at an Au NS dose of 8 pM for 12 h. An uptake of approximately 90% of Au NSs was estimated by TEM and from the removed culture media (see Materials and Methods). Figure 1c shows the relatively high viability of the incubated cells during a period of 19 days, where no depletion of the cell activity was observed.

**Figure 2:**
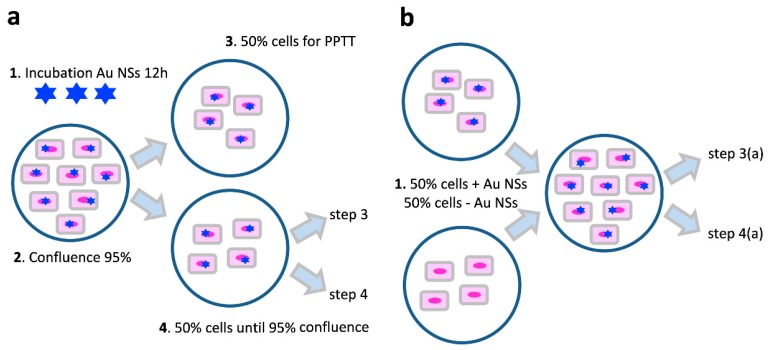
(**a**) Approach one: 1. Incubation of Au NSs for 12 h with uveal melanoma cells. 2. When the culture reached 95% confluence, the cells were divided in two different stocks. 3. The first stock with 50% of the cells was used for laser experiments. 4. The second stock with the other 50% of the cells was left in culture until 95% confluence, at which point it was again divided in two equal stocks to repeat the process. (**b**) Fresh cells and Au NS-loaded cells (8 pM) were mixed at a 1:1 ratio (i.e., Au NS effective concentration of ~4 pM). When the culture reached 95% confluence, it was split in two as explained above (steps 3 and 4) and the process was repeated five times.

To understand the intercellular trafficking properties of Au NSs and their impact on PPTTs, two different sets of experiments were designed (Figure 2). The first strategy consisted of the incubation of uveal melanoma cells with Au NSs at different concentrations for 12 h (Figure 2a). After incubation and washing, and once 95% confluence had been reached, the cell culture was divided in two different stocks with approximately 50% of the cells. While the first stock was used in PPTT experiments, the second one was left in culture until 95% confluence. Then, the second stock was divided again in two equal stocks to repeat the previous step. This strategy was repeated five times until six consecutive cell cultures were obtained. In a second strategy, fresh cells and Au NS-loaded cells were incubated together at a 1:1 ratio (Figure 2b). Once the culture reached 95% confluence, the first strategy was followed again until six consecutive cell cultures were obtained.

**Figure 3:**
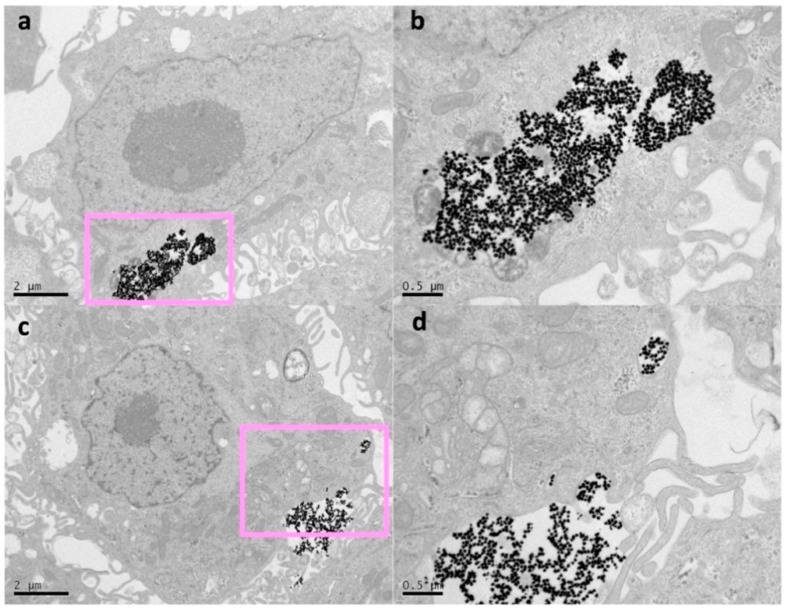
(**a**) TEM micrograph of a single cell loaded with Au NSs and (**b**) image at higher magnification after incubation with 8 pM Au NSs. (**c**) Micrograph after 48 h of incubation and (**d**) image at higher magnification, in which many regions can be seen where the Au NSs have been exocyted and locate between two cells, as well as small vesicular compartments with smaller amounts of Au NS.

Regarding the first strategy, the TEM analysis confirmed that cells were able to uptake, release, and re-uptake Au NSs, where, on average, the number of Au NSs was divided in half during cell division. As representative TEM micrographs, Figure 3a,b show a single cell loaded with a large amount of Au NSs (see also Figures S1 and S2) after 12 h of incubation at an Au NSs dose of 8 pM. On the contrary, after 48 h of incubation, the TEM images show many intercellular regions of exocytosis with non-agglomerated Au NSs (Figure 3c,d). Interestingly, small vesicular compartments of Au NSs inside the cells were observed around the exocytosis regions (see Figure S3). These observations are consistent with previous studies that revealed the dilution and re-endocytosis of Au NPs during cell division [23].

**Figure 4:**
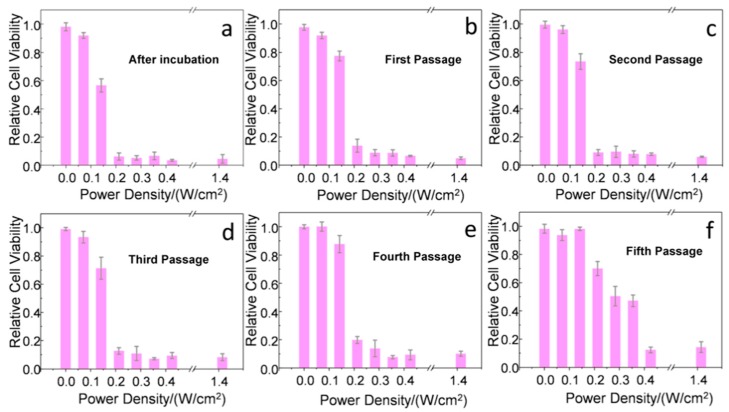
Cell viability as a function of the applied fs laser power density (3 independent samples were tested and averaged at each power density; the error bars show the standard deviation of the relative cell viability). (**a**) After incubation with Au NSs, we observed an important killing rate using laser power densities above 0.21 W/cm^2^. Similar results were obtained after the first (**b**), second (**c**), third (**d**), and fourth (**e**) cell passage, with small differences of 10–20% of cell survival. After the fifth passage (**f**), a laser power density of at least 0.42 W/cm^2^ was required to obtain significant cell killing rates.

In addition, photothermolysis of the cancer cells at different division stages was evaluated by fs laser irradiation at different power densities (from 0 to 1.41 W/cm^2^), maintaining a relatively high exposure surface (20 mm^2^) and low irradiation time (1 min). Right after incubation with the Au NSs (Figure 4a), an important killing rate was observed using laser power densities equal to or above 0.21 W/cm^2^. These power densities are in good agreement with previous results observed for single gold nanorods stabilized with analogous PEG-SH ligands and lower plasmonic efficiencies at the NIR [18]. Very similar results were observed from the first to the fourth cell passage (Figure 4b–e), with small differences of 10–20% in cell survival upon applying a laser power density of 0.21 W/cm^2^ (below the MPE threshold of skin). Interestingly, at the fifth passage, power densities of at least 0.42 W/cm^2^ were required to reach similar cell killing rates (Figure 4f). Therefore, we conclude that an Au NS dilution effect is, in principle, triggered by cell division, considering that exocytosis and re-uptake are able to maintain the Au NS concentration approximately constant. At this point, the Au NS concentration was estimated to be 180 ± 100 Au NS/cell by TEM analysis (see Section 4 and Figure S4).

To corroborate such an Au NS dilution effect, we repeated the same set of experiments with cells incubated at a lower Au NS concentration (2 pM). As expected, a reduction of the PPTT effectiveness was observed in the fourth passage, where power densities of at least 0.42 W/cm^2^ were required to obtain significant cell killing rates (Figures S5 and S6). Due to the proximity between the PPTT effectiveness between irradiations at 2 and 8 pM (three and four cell passages, respectively), no intermediate concentrations of Au NSs were considered. Additionally, we did not observe significant differences of the intercellular regions of endo/exocytosis with the concentration of Au NSs, only related to the amount of loaded nanoparticles at these locations (Figure 3 and Figure S7).

**Figure 5:**
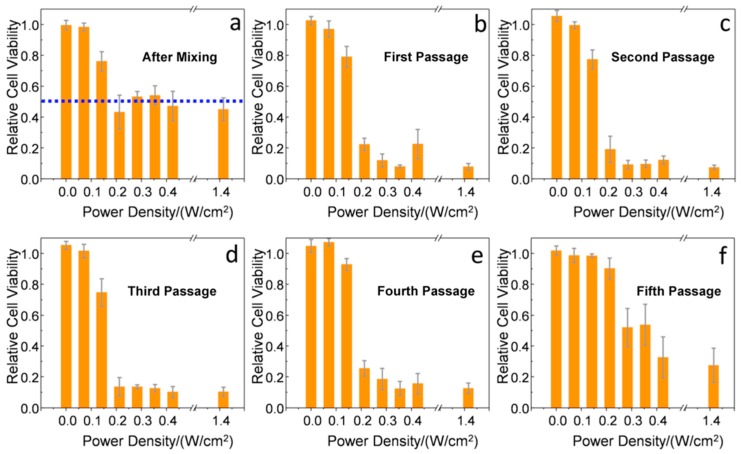
Cell viability as a function of the applied fs laser power density (3 independent samples were tested and averaged at each power density; the error bars show the standard deviation of the relative cell viability). (**a**) After mixing 50% of Au NS-loaded cells and 50% of non-loaded cells, we observed a killing rate of ca. 50% (see blue dashed line) using laser power densities above 0.21 W/cm^2^. After the first (**b**), second (**c**), third (**d**), and fourth (**e**) cell passage, the cell killing rate increased with small differences (10–20%) in cell survival using a laser power density of at least 0.21 W/cm^2^. After the fifth passage (**f**), we did not observe important cell killing rates.

In our second approach (Figure 2b), Au NS-loaded cells incubated at an NP concentration of 8 pM were mixed with fresh cells at a 1:1 ratio, affording an effective concentration of Au NSs of 4 pM. After 8 h of mixing, and using the same laser exposure conditions described before, only around 50% of the cells were killed by the PPTT treatment (Figure 5a), showing that not enough time had passed to allow for intercellular trafficking. However, after the first passage (72 h), an enhanced killing rate of around 85% was observed using a laser power density of 0.21 W/cm^2^ (Figure 5b). This result indicates that, after uptake and exocytosis processes, the Au NSs are re-endocyted by the initially non-loaded cells with retention of their photothermal properties. From the second to the fourth cell passage (Figure 5c–e), the results are very similar with small differences ranging 10–20% of cell survival at a laser power density of 0.21 W/cm^2^. After the fifth passage, as observed in the case of the first strategy at low Au NS concentrations, a reduction of the photothermal efficiency was recorded (Figure 5f). In this case, a laser power density of at least 1.42 W/cm^2^ was required to reach significant cell killing rates (~70%). Therefore, in this second approach, we were able to combine the dilution effect of cell division with a dilution effect upon mixing loaded and non-loaded cells. We estimated the Au NS concentration by TEM analysis to be 3800 ± 900 Au NS/cell after 72 h of mixing (Figure S8) and 90 ± 50 Au NS/cell after the fifth passage (Figure S9).

**Figure 6:**
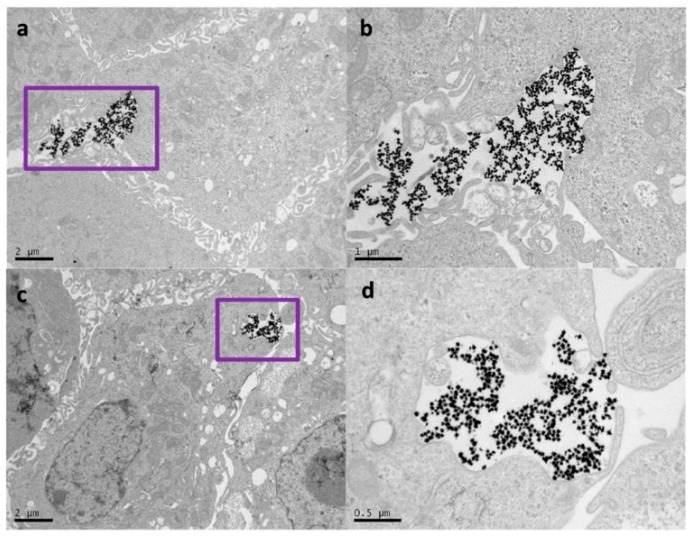
(**a**) TEM micrograph of a single cell where a big membrane invagination can be observed and (**b**) image at higher magnification. Another example of the exocytosis and re-endocytosis of Au NSs is shown in (**c**) and at higher magnification in (**d**).

We further investigated our second approach by TEM analysis. After 24 h, the TEM images showed membrane invaginations in many cells, indicating exocytosis from the Au NS-loaded cells and re-endocytosis by the non-loaded cells (Figure 6). This observation indicates that the Au NS concentration splits during cell division (as manually performed in our first approach), but also by trafficking from loaded cells to non-loaded cells.

## 3. Conclusions

We have studied changes in photothermal effects in regard to the dilution of Au NSs induced by (i) cell division and (ii) by trafficking between Au NS-loaded and non-loaded uveal melanoma cells. In the first approach, we obtained high PPTT efficiency after four cell-division cycles at an initial Au NS concentration of 8 pM. In the second approach, we observed trafficking of Au NSs between loaded and non-loaded cells, achieving effective PPTT after four division cycles under the same low irradiance conditions. Our study reveals the ability of uveal melanoma cells to release and re-uptake Au NSs with retention of their plasmonic photothermal properties throughout several cell-division cycles and after cellular re-uptake. These approaches have the potential for use in real tissue and even to treat the eye tumor itself in the near future. We believe that our method may be used as a co-therapy to disperse plasmonic gold nanostructures across affected tissues, thus increasing the effectiveness of classic PPTT.

## 4. Materials and Methods

Materials. All analytical grade reagents, metallic salts, buffers, and cell culture media were purchased from Sigma-Aldrich or Merck (Madrid, Spain). We used Milli-Q deionized water from a Millipore system (>18 MΩ, Milli Q) in all the experiments.

Optical Characterization. UV-Vis-NIR characterization was performed on a JASCO-V770 spectrophotometer.

Au NSs Synthesis. Au NSs (50.2 ± 4.0 nm analyzed by TEM; LSPR band centered at ca. 800 nm) were prepared by a modified colloidal seed-mediated growth method [31]. Firstly, to prepare the seed solution, 5 mL of 1% citrate solution was added to 100 mL of boiling 0.45 mM HAuCl_4_ solution under vigorous stirring. The seeds (14.0 ± 1.0 nm determined by TEM; 14.9 ± 1 nm measured by Dynamic Light Scattering (DLS); zeta potential of −11.8 ± 0.6 mV) were citrate-stabilized after 15 min of boiling (red color). After the seed solution was cooled down to room temperature, 100 µL of the seed solution was added under gentle stirring to 20 mL of HAuCl_4_ (0.25 mM) solution, containing 20 μL of 1.0 M HCl solution. Following, 200 μL of 3 mM AgNO_3_ and 100 μL of 100 mM ascorbic acid solutions were added simultaneously, and 500 μL of 0.1 mM PEG-SH (6 kDa) solution was added and mixed during 30 min. Finally, Au NSs (56 ± 6 nm measured by DLS; zeta potential of −21 ± 1 mV) were washed by centrifugation at 1190 g and 10 °C for 25 min, and redispersed in Milli-Q water.

Zeta Potential and DLS. The phase analysis light scattering technique (Zeta PALS, Brookhaven Instruments Corp., Holtsville, NY, USA) was employed to measure the electrophoretic mobility, which was then used to obtain the zeta potential of the nanoparticles. The nanoparticle size was determined by a DLS method using a particle analyzer (Zeta Nano Series; Malvern Instruments, Barcelona, Spain). In both studies, samples were determined under experimental conditions of 25 °C, a dispersant refractive index of 1.33 (water), a viscosity of 0.9 cP, and a dispersant dielectric constant of 78.5. Each zeta potential and particle size data point was taken as the average of 50 and 30 independent measurements, respectively.

Cell Culture and Viability. The primary uveal melanoma cell culture, established from a human uveal melanoma primary tumor [33], was cultured in Dulbecco’s modified Eagle’s medium (DMEM) supplemented with 10% FBS, 500 UI/mL penicillin, and 0.1 mg/mL streptomycin, and maintained at 37 °C in 5% CO_2_ in a humidified incubator until confluence. The cell viability was evaluated by the Alamar Blue assay (commercial kit from Life Technologies). Following the guidelines of the manufacturer, the cells were incubated in a 96-well plate with 10% Alamar Blue in DMEM without red phenol for 3 h. The absorbance at 570 nm was monitored using 600 nm as the reference wavelength. The viability was determined by comparison with control cells (100%). All reported experiments were performed in triplicate.

Plasmonic Photothermal Therapy. Photothermal irradiation was performed using a pulsed laser system Ti:sapphire ultrafast oscillator (Tsunami, Spectra-Physics) centered at 800 nm, with a pulse duration of around 90 fs and a repetition rate of 80 MHz. The laser power density was controlled by a variable neutral density filter. Au NSs were incubated at concentrations from 2 and 8 pM for 12 h, after which the cells were washed twice with phosphate buffered saline (PBS) buffer and fresh supplemented DMEM was added. The incubated cells contained in a 96-well plate (7 × 10^3^ cells per well) were illuminated for 1 min with a laser spot diameter of 5 mm. The laser power was evaluated from 0 to 1.41 W/cm^2^. After irradiation, the cells were incubated in a 96-well plate with 10% Alamar Blue in DMEM without red phenol for 3 h following the manufacturer guidelines. Three independent samples were tested and averaged at each power density.

Transmission Electron Microscopy (TEM). TEM images were obtained on a JEOL JEM-1010 transmission electron microscope operating at an acceleration voltage of 80 kV (CNME, Spain). The cells were incubated with the Au NSs, washed twice with PBS buffer, and fixed with a solution containing 2% glutaraldehyde in PBS. Then, the cells were stained with a mixture of 1% osmium tetroxide and 1.5% potassium cyanoferrate. After two washing cycles, the cells were gradually dehydrated in acetone. The samples were embedded in Epon, sectioned for analysis, and cut by ultramicrotomy for observation. The Au NS uptake levels were estimated from TEM images following a procedure described in the literature [18].

## Supplementary Materials

The following are available online at https://www.mdpi.com/2079-4991/10/3/590/s1, Figures S1–S5: TEM micrographs, Figure S6: Cell viability, Figures S7–S9: TEM micrographs.
