# Supplementary material for: Intercellular Trafficking of Gold Nanostars in Uveal Melanoma Cells for Plasmonic Photothermal Therapy
Source: Nanomaterials (Basel). 2020 Mar 24;10(3):590. doi: 10.3390/nano10030590 (PMC7153714; doi:10.3390/nano10030590)
Supplement: Supplementary file 1 [file nanomaterials-10-00590-s001.pdf]

Supporting Information for

Intercellular Trafficking of Gold Nanostars in Uveal Melanoma Cells for Plasmonic Photothermal Therapy

Rubén Ahijado-Guzmán [1,*], Natalia Sánchez-Arribas [1], María Martínez-Negro [1], Guillermo González-Rubio [1], María Santiago-Varela [2], María Pardo [2], Antonio Piñeiro [2], Iván López-Montero [1], Elena Junquera [1] and Andrés Guerrero-Martínez [1,*]

[1] Departamento de Química Física, Universidad Complutense de Madrid, Avenida Complutense s/n, 28040 Madrid, Spain

[2] Instituto de Investigación Hospital Santiago de Compostela (IDIS), Travesía da Choupana s/n Santiago de Compostela, Spain

* Correspondence: rahijado@ucm.es, aguerrero@quim.ucm.es; Tel.: +349344274 (A.G.M.)

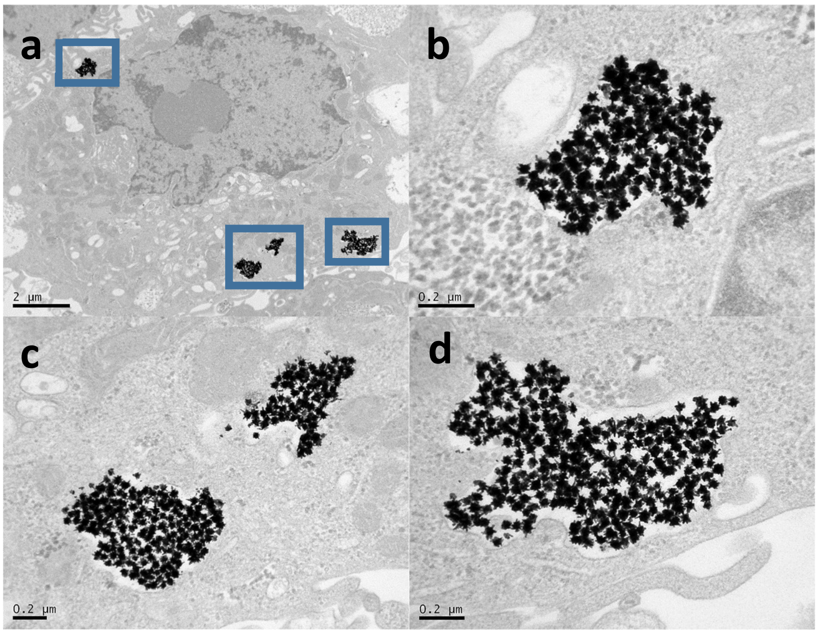

Figure S1. TEM micrographs of (a) single cell loaded with GNS (b, c and d) at higher magnification after the incubation with a GNS dose of 8 pM.

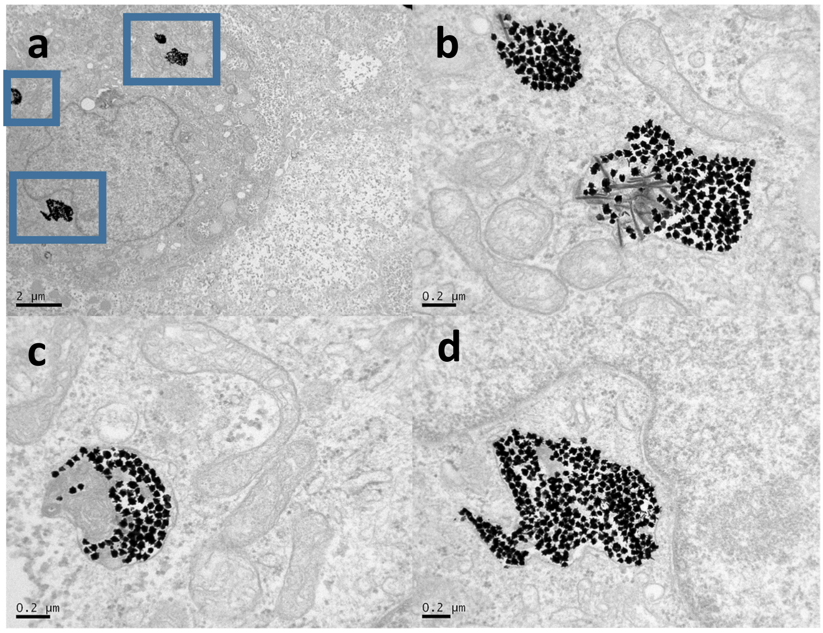

Figure S2. TEM micrographs of (a) single cell loaded with GNS (b, c and d) at higher magnification after the incubation with a GNS dose of 8 pM.

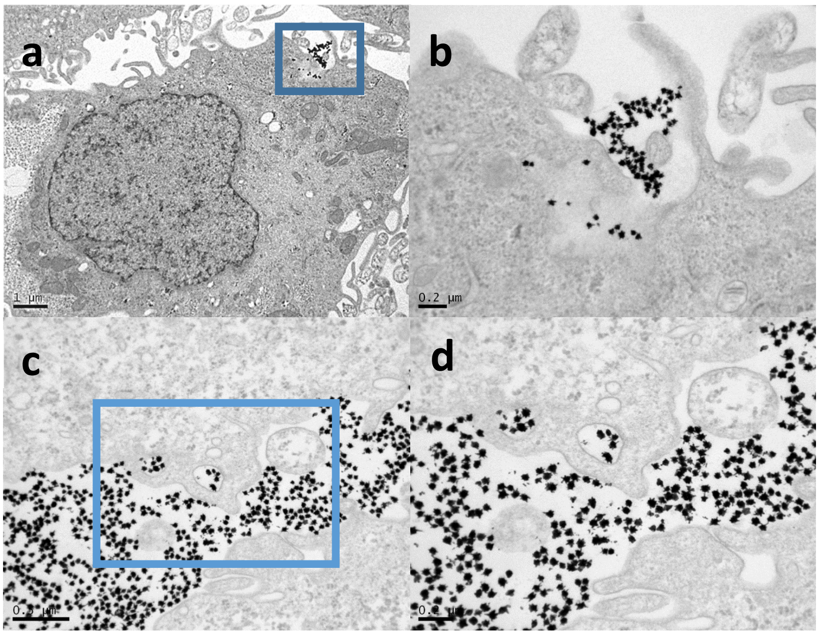

Figure S3. TEM micrographs after 48 hours from the incubation of (a) single cell loaded with GNS (b) at higher magnification after the incubation with a GNS dose of 8 pM. (c) and (d) at higher magnification of a detail between two different cells.

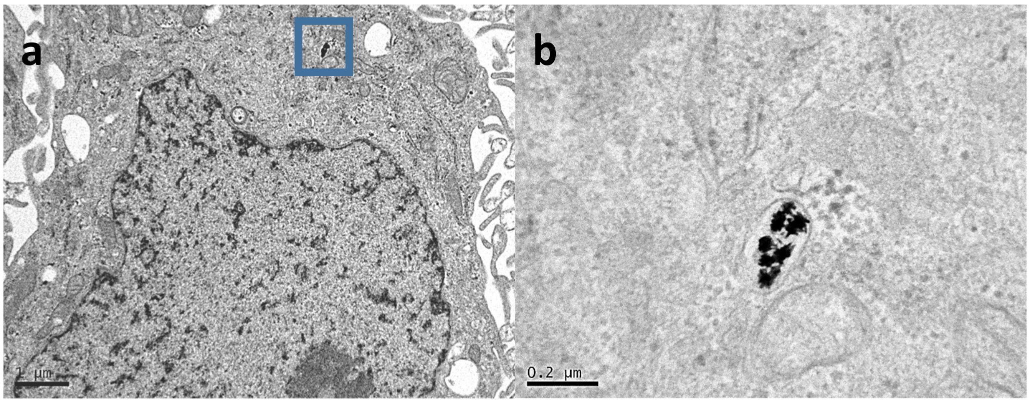

Figure S4. TEM micrographs after 5 passages from the incubation of (a) single cell loaded with GNS (b) at higher magnification after the incubation with a GNS dose of 8 pM.

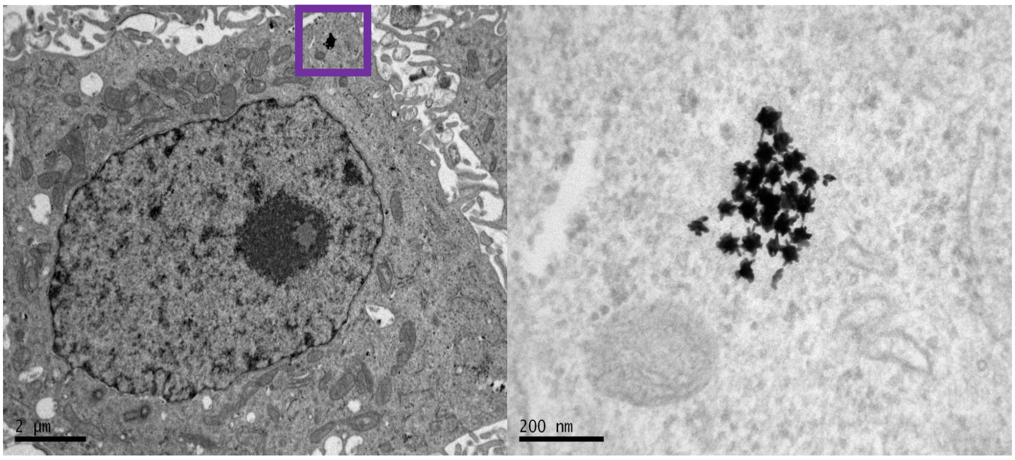

Figure S5. TEM micrographs of (a) single cell loaded with GNS (b, c and d) at higher magnification after the incubation with a GNS dose of 2 pM.

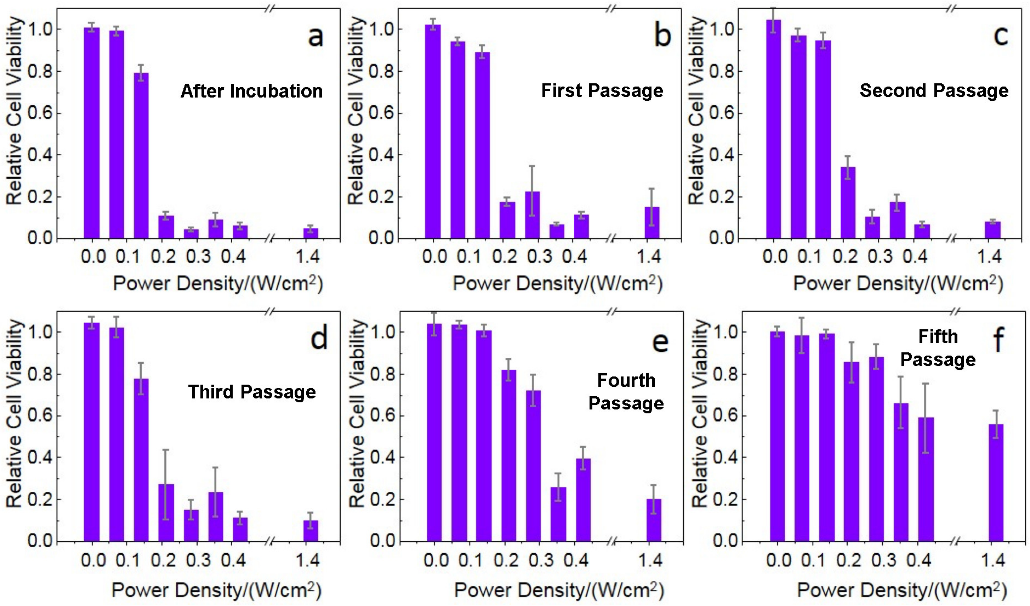

Figure S6. Cell viability as a function of the applied fs laser power density (3 independent samples were tested and averaged at each power density; the error bars show the standard deviation of the relative cell viability). (a) After the incubation with the GNS (2 pM), we observed an important killing rate by using laser power densities higher than 0.21 W/cm^2. Similar result was found after the first (b), the second (c), the third cell passage (d). After the fourth passage (e and f), we did not obtain important cell killing rates.

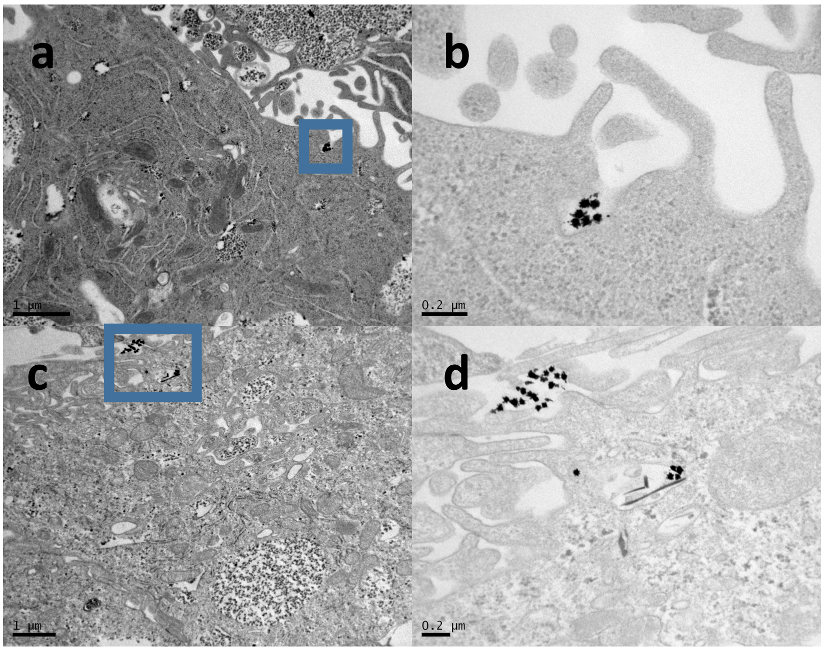

Figure S7. TEM micrographs of single cells after 48 h of incubation with 2 pM Au NSs (a and c), and images at higher magnification, respectively (b and d), in which regions can be seen where the Au NSs have been exocyted and locate between two cells, as well as small vesicular compartments with smaller amounts of Au NS.

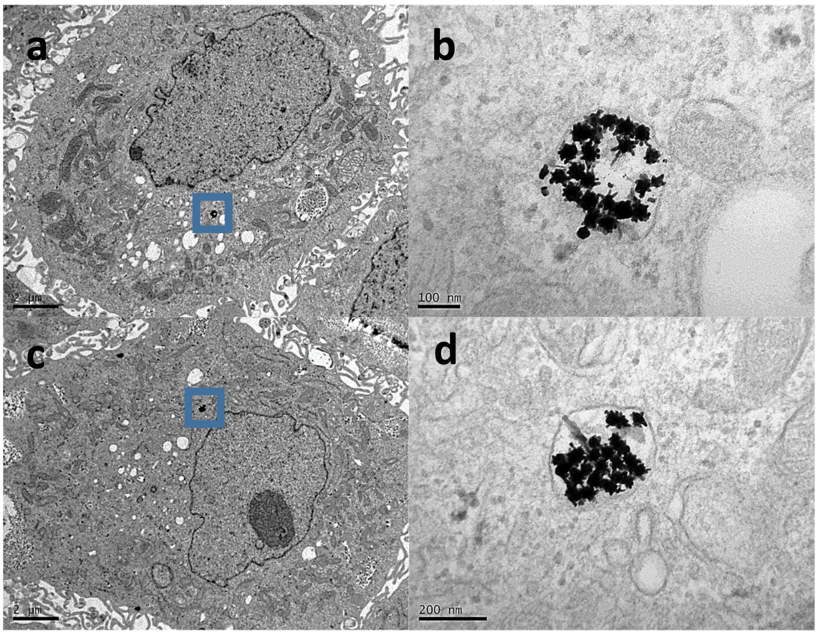

Figure S8. TEM micrographs of (a, c) single cell, (b, d) at higher magnification after 72 hours from the mixing of GNS-loaded and non-loaded cells. Effective GNS dose of 4 pM.

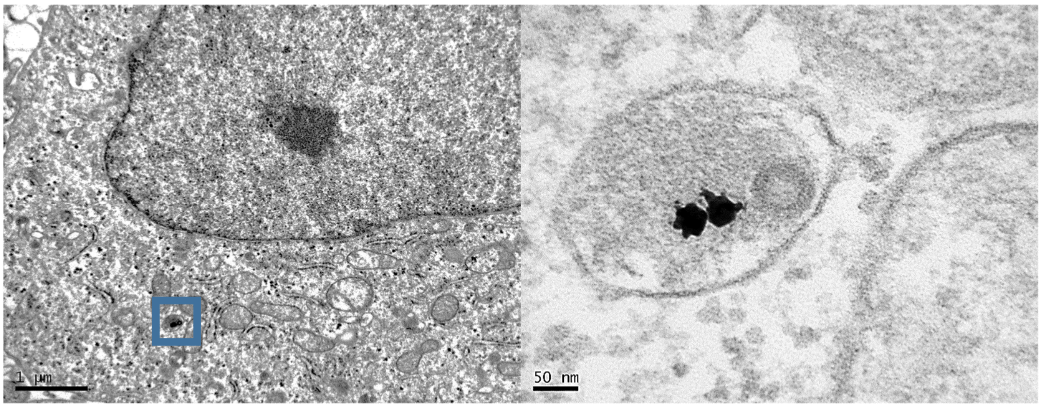

Figure S9. TEM micrographs after 5 passages from the mixing of GNS-loaded and non-loaded cells of (a) single cell (b) at higher magnification.
